# A GX_2_GX_3_G motif facilitates acyl chain sequestration by *Saccharomyces cerevisiae* acyl carrier protein

**DOI:** 10.1016/j.jbc.2021.101394

**Published:** 2021-11-09

**Authors:** Garima, Rashima Prem, Usha Yadav, Monica Sundd

**Affiliations:** NMR Lab, National Institute of Immunology, New Delhi, India

**Keywords:** acyl carrier protein, chemical shift perturbations, NMR, acyl-ACP interaction, fatty acid biosynthesis, acyl chain sequestration, type I ACP, type I FAS, *S. cerevisiae* ACP, 4′-PP, 4′-phosphopantetheine, ACP, acyl carrier protein, CoA, coenzyme A, DSS, sodium 4,4-dimethyl-4-silapentanesulfonate, ESI-MS, electrospray ionization–mass spectrometry, FAS, fatty acid synthesis, HSQC, heteronuclear single quantum coherence spectroscopy, NMR, nuclear magnetic resonance, *Sc*ACP, *Saccharomyces cerevisiae* acyl carrier protein, SEC, size-exclusion chromatography

## Abstract

*Saccharomyces cerevisiae* acyl carrier protein (*Sc*ACP) is a component of the large fungal fatty acid synthase I (FAS I) complex. *Sc*ACP comprises two subdomains: a conserved ACP domain that shares extensive structural homology with other ACPs and a unique structural domain. Unlike the metazoan type I ACP that does not sequester the acyl chain, *Sc*ACP can partially sequester the growing acyl chain within its hydrophobic core by a mechanism that remains elusive. Our studies on the acyl-*Sc*ACP intermediates disclose a unique ^188^GX_2_GX_3_G^195^ sequence in helix II important for ACP function. Complete loss of sequestration was observed upon mutation of the three glycines in this sequence to valine (G188V/G191V/G195V), while G191V and G188V/G191V double mutants displayed a faster rate of acyl chain hydrolysis. Likewise, mutation of Thr216 to Ala altered the size of the hydrophobic cavity, resulting in loss of C_12_- chain sequestration. Combining NMR studies with insights from the crystal structure, we show that three glycines in helix II and a threonine in helix IV favor conformational change, which in turn generate space for acyl chain sequestration. Furthermore, we identified the primary hydrophobic cavity of *Sc*ACP, present between the carboxyl end of helix II and IV. The opening of the cavity lies between the second and third turns of helix II and loop II. Overall, the study highlights a novel role of the GX_2_GX_3_G motif in regulating acyl chain sequestration, vital for *Sc*ACP function.

Fatty acid biosynthesis is an important cellular process, indispensable for membrane biogenesis, cell metabolism, and generation of signaling molecules. Based on the organization, fatty acid biosynthetic machinery has been categorized into (a) dissociated type (type II pathway) observed in prokaryotes, plastids, and mitochondria; and (b) type I pathway, found in eukaryotes and a few bacterial species. Each catalytic unit is an independent protein in the type II pathway. On the contrary, type I pathway enzymes exist as domains of a large, multifunctional fatty acid synthase (FAS). Within the type I FAS, two different subtypes have been identified: a 540 kDa homodimer present in metazoans, while in yeast/fungi/actinomycetes, a 2.6 MDa dodecameric (α6β6) complex is present. The complex forms a barrel-shaped structure with six β-subunits, three on either side of a central disc formed by six α-subunits (1, 2). Seven catalytically active centers and the ACP domain may be present on a single polypeptide (*e.g.*, *Rhodosporidium toruloides*) or two different polypeptides (*e.g*., *Saccharomyces cerevisiae*) (2, 3).

Acyl carrier protein (ACP) is an indispensable component of both type I and type II FAS. It has a four-helix bundle fold, with a conserved serine that tethers a coenzyme-A derived 4′-phosphopantetheine moiety (4′-PP). The free sulfhydryl group of the 4′-PP arm anchors the growing acyl chain by a thioester bond. All type II ACPs can sequester the lengthening acyl chain deep into their hydrophobic core. The well-studied ones are *Escherichia coli*, spinach, *Plasmodium falciparum*, *Helicobacter pylori* (4, 5, 6, 7), *Leishmania major* (8), and *Streptomyces coelicolor* (9). *E. coli* acyl-ACP crystal structures show that the aliphatic chain remains buried in the hydrophobic core, interacting with the side chain of hydrophobic residues lining the cavity. The labile thioester linkage between 4′-phosphopantetheine and the acyl chain also remains embedded in the cavity, and the terminal methyl group of the aliphatic-chain points toward the C-terminus of helix II and IV (4). Helix I, III, and the N-terminus of helix II move outward in C_10_-*Ec*ACP, to accommodate the growing acyl chain (10). The conformation of butyryl (C_4_-), hexanoyl (C_6_-), and heptanoyl (C_7_-) ACP structures lies between the unliganded and decanoyl (C_10_)-ACP conformations (4, 10). Similarly, in spinach ACP, the backbone experiences a major conformational change to sequester the lengthening acyl chain (5). A switchblade mechanism transfers the acyl chain from the hydrophobic cavity of ACP to the active site of the FAS enzymes (11). Acyl chain flipping has been observed in the crystal structure of *E. coli* ACP-FabA (fatty acid 3-hydroxyacyl-ACP dehydratase) cross-linked complex (12). Interestingly, the type II FAS enzymes can discriminate between acyl-ACP intermediates *via* allosteric interactions, even without flipping the acyl chain (13).

Type I ACP (metazoan) differs from type II in their overall surface charge and hydrophobicity. NMR studies on rat ACP demonstrate that the acylated forms do not experience significant chemical shift perturbations, suggesting lack of chain sequestration in the central cavity. Type I ACP also lacks the chain flipping mechanism. Instead, the phosphopantetheine-bound acyl chain remains buried within the grooves, present on its hydrophobic surface (14). *In silico* studies highlight the role of bulky hydrophobic residues present between helix II and III, *i.e.*, L2144, L2146, and V2174 in locking the ACP in a closed state. The modular and compact organization of reaction centers in type I FAS probably obviates the need for acyl chain sequestration (1).

Fungal FAS type I complex (PDB 2UV8) is remarkably different from the rat/pig type I system (PDB 2VZ8) in its overall size and architecture, revealed by crystallography and cryo-EM studies (2, 15). In addition to the canonical ACP fold, its ACP domain contains a four-helix subdomain that stabilizes the ACP structure. This class of type I ACP can sequester acyl chains as long as decanoyl (2, 16). The mechanism underlying acyl chain sequestration by fungal ACP and the delivery of acyl cargo to the cognate enzymes is not yet well understood. Progress in elucidating the same has been limited due to the large ACP didomain size (∼20 kDa) and short shelf life of the intermediates.

Furthermore, fungal *FAS2* gene product has been recognized as a candidate target for drug intervention in *Candida parapsilosis* and *Candida albicans.* The microorganism depends on fatty acids for growth and virulence (17, 18). In view of the rising number of invasive fungal infections in immunosuppressed patients, and the emerging problem of drug resistance, it is necessary to study fungal FAS domains at the molecular level. *S. cerevisiae* is a widely accepted model to understand lipid metabolism in eukaryotes. Multiple X-ray (PDB 2UV8, 6QL9) and cryo-EM structures (PDB 6U5U, 6TA1) are available for its FAS complex (2, 15). NMR assignments of its ACP domain have also been published (19). Therefore, we have used *S. cerevisiae* ACP as a prototype, to understand acyl chain sequestration by fungal FAS. Our studies uncover a unique arrangement of glycine’s in helix II and a threonine in helix IV that play crucial role in acyl chain sequestration. Moreover, we have identified the hydrophobic cavity of *Sc*ACP that sequesters the growing acyl chain.

## Results

**Figure 1:**
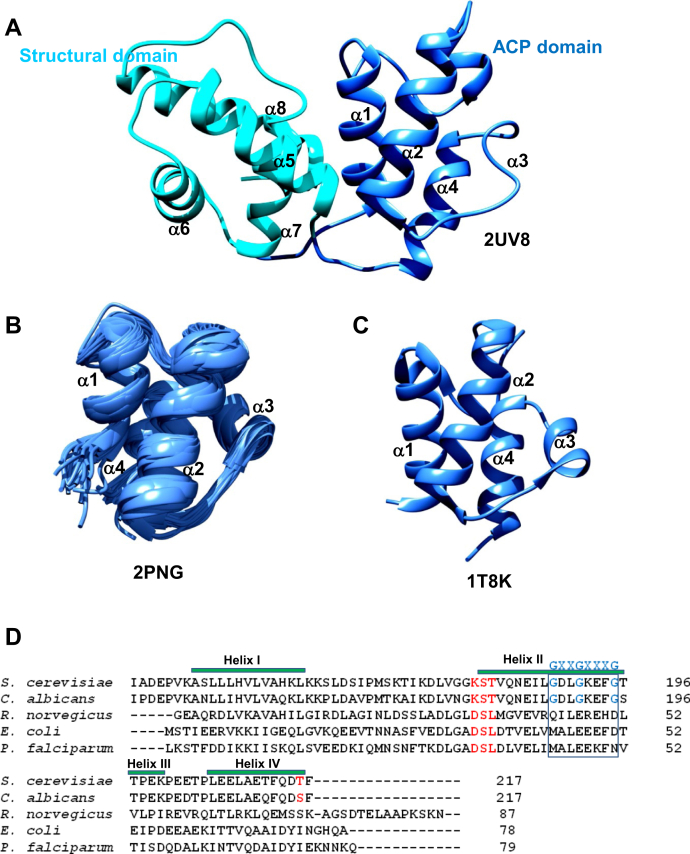
**The ACP subdomain of *Sc*ACP is homologous to other type I and type II ACPs.** A ribbon diagram of (*A*) *Sc*ACP didomain, displaying the ACP subdomain *blue*, and the structural domain cyan. *B*, type I ACP of rat (PDB 2PNG, lowest 20 NMR models), and (*C*) type II ACP of *E. coli* (PDB 1T8K), shown as ribbons. The figure was prepared using UCSF-Chimera (21). *D*, sequence comparison of *Sc*ACP (ACP subdomain) with other type I and type II ACPs. A GX_2_GX_3_G sequence is indicated by a *blue box*, conserved only in *Saccharomyces* and *Candida* spp. Sequence alignment was done using Clustal omega (https://www.ebi.ac.uk/Tools/msa/clustalo).

*S. cerevisiae* acyl carrier protein (*Sc*ACP) comprises two sub domains, (a) a canonical ACP domain (Ala138-Gly219) homologous to most type I and type II ACPs, and (b) a structural domain (Ala220-Leu302) found in fungi and a few bacterial species. Figure 1*A* shows a ribbon representation of the *Sc*ACP didomain obtained from the full-length FAS structure (PDB 2UV8). The NMR structure of *Sc*ACP is also available, PDB 2ML8 (16). In both the structures, the ACP subdomain (colored blue in Fig. 1*A*) comprises helices I–IV, and the structural domain is formed by helices V–VIII (colored cyan). The ACP subdomain has a four-helix bundle fold, similar to the type I ACP of rat (PDB 2PNG) and type II ACP of *E. coli* (PDB 1T8K), shown in Figure 1, *B* and *C*. The α1-α4 helices of *Sc*ACP are analogous to the four helices of other type I and type II ACP (2). *Sc*ACP helix III displays a turn between Lys 200 and Thr 204, i + 4 residues apart in the solution structure (PDB 2ML8) (16). VADAR identified a backbone hydrogen bond at the same position in the crystal structure (2UV8), though Figure 1*A* generated using Chimera does not display a helical turn in that region (20, 21). Figure 1*D* shows the multiple sequence alignment of *Sc*ACP subdomain with *C. albicans*, *R. norvegicus*, *E. coli*, and *P. falciparum* ACP. The conserved DSL motif of most type I and II ACPs is substituted with a KST sequence in *Sc*ACP. Met 44 of type II ACP is replaced with a Gly, and ^44^MALEEEFDTE^53^ helix II sequence of *E. coli* ACP corresponds to a unique glycine rich motif ^188^GDLGKEFGTT^197^ of *Sc*ACP. Residue numbering used in this study is in accordance with the available NMR (PDB 2ML8) and crystal structures (2UV8) (1, 16).

### Acylation-induced chemical shift perturbations of *Sc*ACP are characteristic of chain sequestration

Uniformly labeled [^1^H^15^N^13^C] apo-*Sc*ACP didomain (Ala 138-Leu 302) was enzymatically converted into various acyl-intermediates using its cognate 4′-phosphopantetheinyl transferase *Sc*PPT (UNP P19097, Asn 1755-Gln 1877) and the respective acyl-coenzyme A. Conversion of the apo-form to the acyl-intermediate was confirmed by (a) native-PAGE, as the acylated form migrates ahead of apo-*Sc*ACP due to a higher negative charge, (b) molecular mass determined by electrospray ionization–mass spectrometry (ESI-MS), and (c) perturbations at the amides of Lys 179, Gln 183, and Asn 184 in a ^1^H^15^N HSQC spectrum, the hallmark of apo-to holo-*Sc*ACP conversion. Holo-*Sc*ACP chemical shifts were assigned based on the BMRB ID 16085 (19). C_4_- (δC_4-_-δHolo-), C_14_- (δC_14-_-δHolo-), and C_16_-*Sc*ACP (δC_16_-δHolo-) ^1^H^15^N HSQC spectra can be fully superimposed on the holo-*Sc*ACP spectra, suggesting lack of chain sequestration (data not shown). In contrast, C_8_- (δC_8_-δHolo-), C_10_- (δC_10_-δC_8_-), and C_12_-*Sc*ACP (δC_12_-δC_10_-) amides displayed significant perturbations in both proton (^1^H) and nitrogen (^15^N) dimension. The two nuclei report very different interactions/changes. Proton chemical shifts are influenced by hydrogen bonds, inductive effects, and ring current effects, while nitrogen chemical shifts report secondary structure perturbations, changes in dihedral angles and hydrogen bonds of the preceding carbonyl (22, 23). Therefore, we have reported acylation-induced *Sc*ACP perturbations separately for ^1^H and ^15^N nuclei.

**Figure 2:**
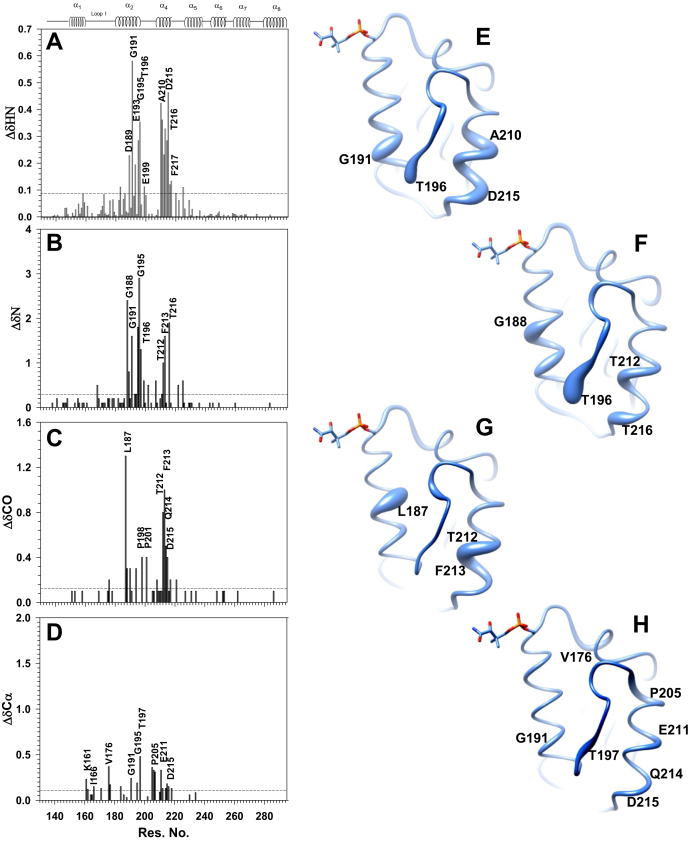
**Chemical shift perturbations suggest C**_**12**_**-chain sequestration by *Sc*ACP.** Changes in the backbone (*A*) amide proton, (*B*) nitrogen, (*C*) carbonyl, and (*D*) Cα chemical shift of *Sc*ACP upon C_12_-intermediate (δC_12_-δHolo-*Sc*ACP) formation. A worm representation of *Sc*ACP, displaying the changes in amide (*E*) proton, (*F*) nitrogen, (*G*) carbonyl, and (*H*) Cα chemical shift as swellings. In each case, the thickness of the worm is directly proportional to the magnitude of chemical shift change. The figure was prepared using UCSF-Chimera (21).

**Figure 3:**
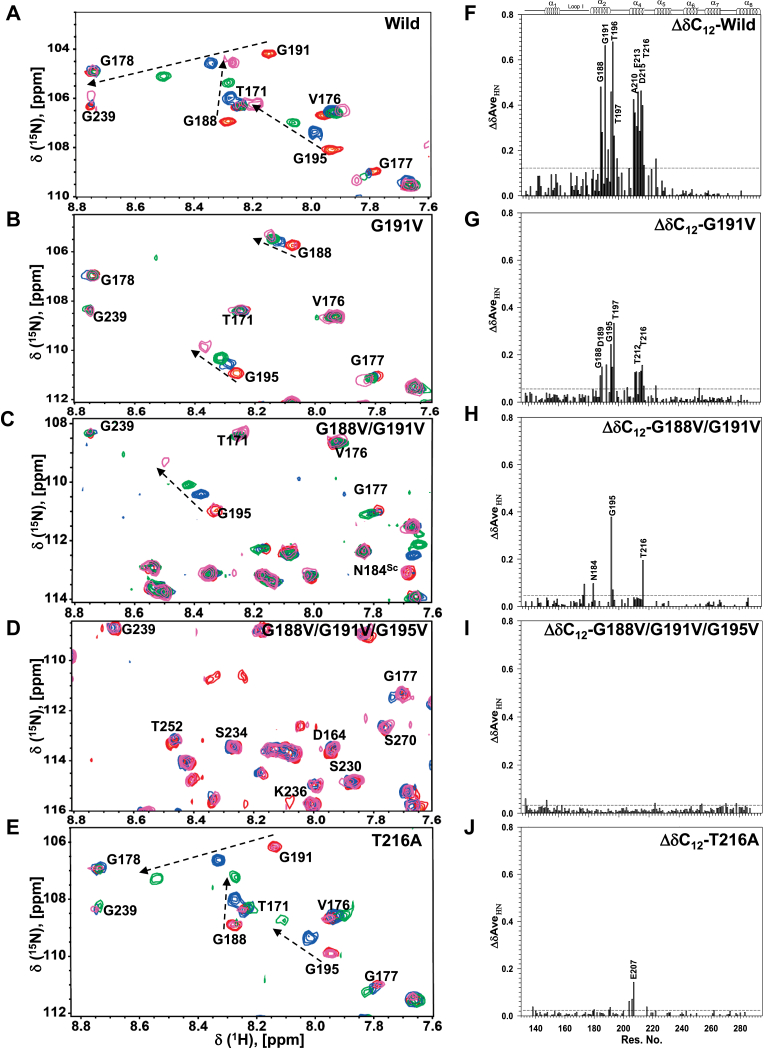
**A GX**_**2**_**GX**_**3**_**G motif in helix II and a threonine in helix IV are necessary for acyl chain sequestration.** Multiple overlaid ^1^H^15^N HSQC spectra for (*A*) Wild, (*B*) G191V, (*C*) G188V/G191V, (*D*) G188V/G191V/G195V, and (*E*) T216A *Sc*ACP, displaying Gly 188, Gly 191, and Gly 195 peaks. Holo-peaks are *colored red*, C_8_- *blue*, C_10_- *green*, and C_12_- *magenta*. Weighted average amide chemical shift changes observed upon formation of C_12_- intermediates of (*F*) Wild, (*G*) G191V, (*H*) G188V/G191V, (*I*) G188V/G191V/G195V and (*J*) T216A *Sc*ACP. For the triple mutant G188V/G191V/G195V, ^15^N TROSY-HSQC spectra were acquired as it gave better signal. One standard deviation is shown as a discontinuous *horizontal line*. ΔδAve_HN_ chemical shifts were calculated using Equation 1.

Figure 2, *A* and *B* display a plot for the changes in ^1^H and ^15^N chemical shift of dodecanoyl-*Sc*ACP (δC_12-_-δHolo-) as a function of residue number. Changes in ^1^H chemical shift were observed for Gln 183, Asp 189, Gly 191, Glu 193, Gly 195, Thr196, Glu 199, Ala 210-Phe 217, and Ser 225, shown in Figure 2*A*. One standard deviation has been used as a cutoff to demarcate significant change. The residues displaying significant perturbation are localized in helix II, loop II, and helix IV, shown as swellings of the worm in Figure 2*E*. The magnitude of chemical shift change is directly proportional to the thickness of the worm. Nitrogen chemical shift changes were observed for Gly 188, Asp 189, Gly 191, Phe 194-Thr 196, Glu 199, Glu 202, Glu 207, Thr 212, Phe 213, Thr 216, Gly 222, and Ser 225. Several of these amides overlap with the ones displaying ^1^H perturbations. The aforementioned residues are present in helix II, loop II, and helix IV, illustrated in Figure 2*F*. Figure 3*A* displays a multiple overlaid ^1^H^15^N HSQC spectra for holo-*Sc*ACP and its intermediates. Change in peak position of Gly188, Gly191 and Gly195 amide is shown. Thr 212 and Thr 216 peaks shifts in the intermediates are illustrated in Figure S1, *A* and *B*, respectively. The direction of chemical shift change is indicated by a forward pointing arrow. Holo peaks are colored red, C_8_- blue, C_10_- green, and C_12_-*Sc*ACP magenta. Same color coding has been used throughout for all the spectra reported in the study. The molecular mass of apo-, holo-*Sc*ACP and acyl intermediates was determined using ESI-MS (Fig. S2, *A*–*F*). The observed mass in each case was within ±1 Da of the expected value.

Figure S3 shows the weighted-average amide chemical shift changes of C_8-_ (δC_8-_-δHolo-), C_10-_ (δC_10-_-δC_8-_), and C_12-_ (δC_12-_-δC_10_-) *Sc*ACP. The average amide chemical shift perturbations (ΔδAve_HN_) were calculated using Equation 1, described under Experimental procedures. The ΔδAve_HN_ changes have been mapped to a worm figure as well (Fig. S3, *D*–*F*). Notably, C_8_-*Sc*ACP displayed perturbations primarily in helix II and loop II (Fig. S3, *A* and *D*). C_10_- *Sc*ACP induced changes in helix II, loop II, and helix IV (Fig. S3, *B* and *E*). C_12_-*Sc*ACP formation was accompanied with large magnitude chemical shift changes of helix II, loop II, and helix IV amides (Fig. S3, *C* and *F*).

Chemical shift changes>1SD were also observed at the carbonyls of several residues of C_12_*-Sc*ACP. Val 176, Leu187, Gly 188, Leu 190, Phe 194, Pro 198, Pro 201, Thr 212, Phe 213, Gln 214, Asp 215, Thr 217, and Leu 221 displayed significant change (Fig. 2*C*). The aforementioned residues are localized in helix II, loop II, and helix IV, as shown in Figure 2*G*. Perturbations are shown as swelling of the *Sc*ACP worm diagram. Figure S1, *C*–*F* display superimposed HNCO spectra for Leu187, Phe 194, Thr 212, and Asp 215, respectively. Large upfield changes were observed for Leu 187 carbonyl (∼1.35 ppm), Thr 212 (∼0.8 ppm), and Phe 213 (∼1.0 ppm). Solid-state NMR studies have previously shown that the chemical shift of a hydrogen-bonded amide proton or carbonyl displays magnetic deshielding with the decrease in R_N…O_ distance (24, 25, 26). Thus, the upfield change in the carbonyl chemical shift of the abovementioned residues indicates lengthening of their associated backbone hydrogen bonds.

Cα chemical shift changes of C_12_-*Sc*ACP (δC_12-_-δHolo-) were also followed as a function of residue number, illustrated in Figure 2*D*. Residues displaying Cα chemical shift change have been mapped to the *Sc*ACP worm structure in Figure 2*H*. Overall, the observed Cα changes were relatively small, primarily in helix II and loop II residues, consistent with a previous NMR study (16).

Taken together, the unique pattern of acyl-*Sc*ACP perturbations, observed simultaneously in helix II, loop II and helix IV, is a hallmark of chain sequestration.

### TALOS+ predicts small changes in the dihedral angles of helix II, loop II, and helix IV of C_12_-*Sc*ACP

**Figure 4:**
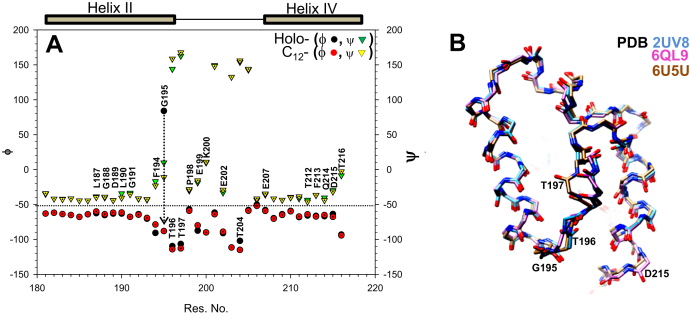
**TALOS+ predicts minor dihedral angle changes in helix II, loop II and helix IV.***A*, a plot of backbone dihedral angles as a function of residue number. The φ,ψ angles for holo-*Sc*ACP are represented by *black filled circles* and inverted *green triangles*, while the symbols for the φ,ψ angles of C_12_-*Sc*ACP are *red filled circles* and *yellow inverted triangles*, respectively. *B*, overlay of the *Sc*ACP didomain backbone in the PDB structures 2UV8 (*cyan*), 6QL9 (*pink*), and 6U5U (*brown*).

^1^H, ^15^N, ^13^CO, and ^13^Cα chemical shifts were used to predict dihedral angles of holo- and C_12_-*Sc*ACP using TALOS+ (27). Figure 4*A* displays a plot for the φ, ψ angles of holo-*Sc*ACP and its dodecanoyl- form, as a function of residue number. In the figure, black filled circles represent φ angles and yellow triangles ψ angles for wild-type *Sc*ACP. C_12_-*Sc*ACP φ angles are shown by red filled circles and ψ angles by green triangles. A dotted line separates the φ and ψ angles. As illustrated in the figure, several residues present in helix II, loop II, and helix IV displayed minor changes in dihedral angles, suggesting slight alteration of the backbone conformation. Though most of the changes are relatively small, added together they may induce a significant change in the size of the hydrophobic cavity. Notably, Gly 195 displayed a large change in its φ angle in C_12_-ScACP compared with wild-*Sc*ACP. Using VADAR, we measured the φ, ψ dihedral angles of Gly 195 in the crystal structure PDB 2UV8 (92°, −43°) and the NMR structure 2ML8 (75.3°, −18°). These values are comparable to the holo-*Sc*ACP dihedral angles determined (84° and 10°) in the present study. However, in C_12_-*Sc*ACP, a noticeable change was observed (−88° and 11°), suggesting a 180° flip of the Gly 195 backbone. These TALOS+ predictions were substantiated by *Sc*ACP crystal (2UV8, 6QL9) and cryo-EM structure (6U5U), where Gly 195 and other loop II residues display a remarkably different conformation (Fig. 4*B*).

### Helanal-Plus detects large bending angles in helices II and IV

Three glycines present within a ^188^GX_2_GX_3_G^195^ motif of helix II displayed significant perturbations in C_12_-*Sc*ACP. Glycine has been shown to induce kinks in helices. Therefore, Helanal-Plus server was used to predict bending angles in the crystal structure PDB 2UV8 (28). Local bending angles were calculated from the helix axes fitted to the Cα atoms of residues (i − 3) − i and i − (i + 3). Table 1 lists the bending angles for helices II and IV of holo-*Sc*ACP determined using Helanal-Plus. Both the helices displayed slight distortions in their axes. A bending angle of ∼10° was observed for the axes fitted to Glu185 Gly188 Gly188 Gly191 and 11° for Gly188 Gly191 Gly191 and Phe194, in helix III. Similarly, in helix IV, a bending angle of 13.6° was observed for the axes fitted to Leu209 Thr212 Thr212 and Asp215. These bending angles are less than 20° and therefore pro-kink. However, they might transform into a kink under certain functional conditions.

**Table 1:** Bending angles calculated from local helix axes fitted to CA atoms ((i − 3) − i) and (i − (i + 3)) using Helanal-Plus (28)

Res. (i − 3)	Res. i	Res. i	Res. (i + 3)	Bending angle (°)
180 S	183 Q	183 Q	186 I	5.6
181 T	184 N	184 N	187 L	2.9
182 V	185 E	185 E	188 G	2.4
183 Q	186 I	186 I	189 D	4.7
184 N	187 L	187 L	190 L	8.5
185 E	188 G	188 G	191 G	10.2
186 I	189 D	189 D	192 K	8.4
187 L	190 L	190 L	193 E	9.5
188 G	191 G	191 G	194 F	11.2
**206 L**	**209 L**	**209 L**	**212 T**	**3.5**
**207 E**	**210 A**	**210 A**	**213 F**	**8.4**
**208 E**	**211 E**	**211 E**	**214 Q**	**6.4**
**209 L**	**212 T**	**212 T**	**215 D**	**13.6**
**210 A**	**213 F**	**213 F**	**216 T**	**8.3**

Helix IV as represented in bold.

### VADAR identifies long/broken hydrogen bonds in helix II and IV

Backbone hydrogen bonds were calculated for the *Sc*ACP didomain, PDB 2UV8 using VADAR (Volume Area Dihedral Angle Reporter), listed in Table 2 (20). In helix II, Leu187CO-NGly191 hydrogen bond is longer than average, ∼2.8 Å. In helix IV, several longer than average hydrogen bonds were identified; Glu208 CO-NThr212 is 2.9 Å, while Glu 211CO-NAsp215 and Thr212CO-NThr216 could not be detected by VADAR. The corresponding CO-N distances were measured manually using Chimera, ∼2.7 Å and 3.4 Å, respectively (21).

**Table 2:** Backbone hydrogen bonds in helix II and IV of *Sc*ACP, (PDB 2UV8) calculated using VADAR (20)

H Bond acceptor (CO)	H Bond donor (N)	O-N distance Å	Type
Thr 181	Glu 185	2.36, 2.34, 2.33	i, i + 4
Val 182	Ile 186	2.35, 2.34, 2.36	i, i + 4
Gln 183	Leu 187	2.21, 2.18, 2.20	i, i + 4
Asn 184	Gly 188	2.32, 2.29, 2.28	i, i + 4
Glu 185	Asp 189	2.07, 2.07, 2.09	i, i + 4
Ile 186	Leu 190	2.39, 2.43, 2.41	i, i + 4
Leu 187	Gly 191	2.80, 2.80, 2.81	i, i + 4
Gly 188	Lys 192	2.56, 2.59, 2.58	i, i + 4
Asp 189	Glu 193	2.63, 2.63, 2.62	i, i + 4
Leu 190	Phe 194	2.04, 2.03, 2.07	i, i + 4
Gly 191	Gly 195	2.34, 2.31, 2.36	i, i + 4
**Leu 206**	**Ala 210**	**2.11, 2.10, 2.08**	**i, i** + **4**
**Glu 207**	**Glu 211**	**2.54, 2.56, 2.54**	**i, i** + **4**
**Glu 208**	**Thr 212**	**2.92, 2.90, 2.87**	**i, i** + **4**
**Leu 209**	**Phe 213**	**2.10, 2.10, 2.10**	**i, i** + **4**
**Ala 210**	**Gln 214**	**2.37, 2.39, 2.39**	**i, i** + **4**
**Glu 211**	**Asp 215**	**Long**	**i, i** + **4**
**Glu 211**	**Gln 214**	**2.60, 2.57, 2.55**	**i, i** + **3**
**Thr 212**	**Thr 216**	**Long**	**i, i** + **4**
**Phe 213**	**Phe 217**	**2.63, 2.57, 2.59**	**i, i** + **4**

The three H bond length values correspond to chains A, B, and C in the crystal structure (PDB 2UV8). Helix IV as represented in bold.

### ^188^GX_2_GX_3_G^195^ sequence is necessary for acyl chain sequestration

To uncover the role of the ^188^GX_2_GX_3_G^195^motif in *Sc*ACP function, Gly 188, Gly 191, and Gly 195 were singly mutated to valine. A G188V/G191V (double mutant), as well as G188V/G191V/G195V (triple mutant), was also generated. Figure S4, *A*–*C* and *F*–*H* show changes in the ^1^H and ^15^N chemical shift upon mutagenesis in G188V, G191V, and G195V*Sc*ACP. Perturbations were observed primarily near the site of mutation, suggesting no major change in the overall fold.

Figure 3, *B*–*D* show regions of multiple overlaid ^1^H^15^N HSQC spectra for holo-, C_8_-, C_10_-, and C_12_-intermediates of G191V, G188V/G191V, and G188V/G191V/G195V, respectively. Figure 3*G* shows a histogram for the average amide chemical shift changes (ΔδAve_HN_) of G191V*Sc*ACP upon conversion to its C_12_- intermediate (δC_12_-δholo-). A remarkable decrease in the magnitude of chemical shift change was observed in C_12_-G191V*Sc*ACP (single mutant), compared with C_12_-wild type *Sc*ACP. C_12_-G188V/G191V*Sc*ACP (double-mutant) sample also displayed a decrease in the magnitude of chemical shift change (Fig. 3, *C* and *H*). In the triple-mutant G188V/G191V/G195V*Sc*ACP, the ^1^H^15^N HSQC spectra of C_8_- (blue), C_10_- green, C_12_- (magenta), and holo-form (red) overlapped completely (Fig. 3*D*). ΔδAve_HN_ chemical shift perturbations were insignificant (Fig. 3*I*), suggesting complete loss of chain engulfment in the G188V/G191V/G195V*Sc*ACP cavity (Fig. 3*F*). The chemical shift of Gln 183 and Asn184 amides corroborated the conversion of apo-to C_12_-*Sc*ACP. The molecular mass of all the intermediates was confirmed by ESI-MS. Due to the hydrophobic nature of the triple mutant, obtaining good-quality ESI-MS spectra for the fully converted C_12_- triple mutant was a challenge. We were able to obtain ESI-MS spectra for the half-converted C_8_- G188V/G191V/G195V*Sc*ACP (Fig. S2*G*). Two species could be identified, (a) C_8_-form (18,615 Da), and (b) apo-form (18,203 Da), confirming the formation of triple mutant intermediates.

**Figure 5:**
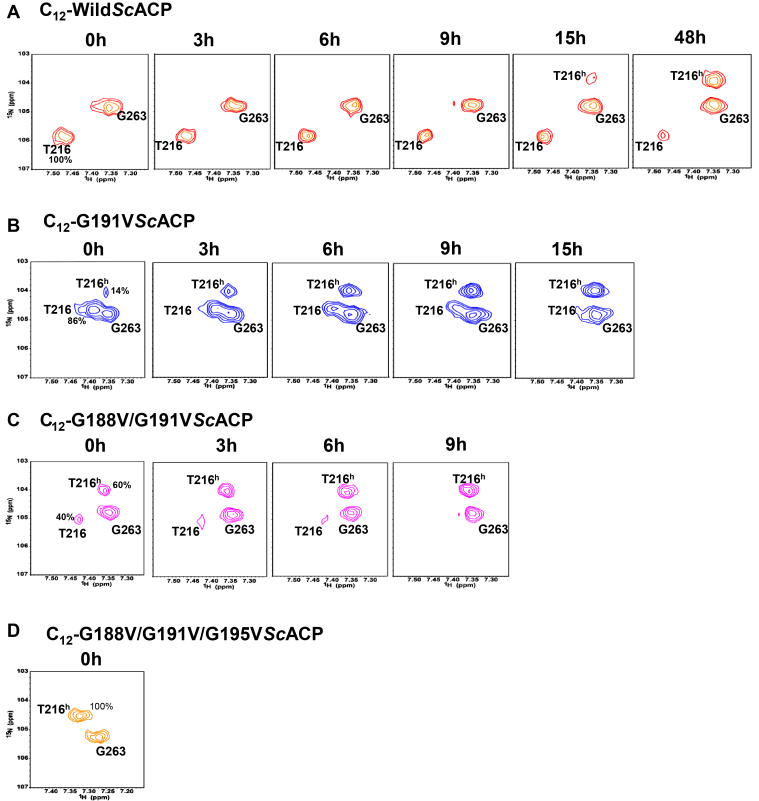
**Mutation of glycines in the GX**_**2**_**GX**_**3**_**G motif increases the rate of acyl chain hydrolysis.**^1^H^15^N HSQC spectra displaying chemical shift changes of Thr216 HN peak in the C_12_- intermediates of (*A*) holo-, (*B*) G191V, (*C*) G188V/G191V, and (*D*) G188V/G191V/G195V*Sc*ACP. Thr216 peak hydrolyzes to the holo-form (T216^h^) over a period of ∼3 days in wild *Sc*ACP at room temperature.

Hydrolysis of the C_12_- chain was followed in wild type, G191V, G188V/G191V, and G188V/G191V/G195V*Sc*ACP dodecanoyl intermediates as a function of time. This was done by acquiring a series of ^1^H^15^N HSQC spectra at 298 K. As illustrated in Figure 5*A*, Thr216 HN has a chemical shift of 106 ppm in wild-type C_12_-*Sc*ACP. In a freshly prepared C_12_-wild-type sample, C_12_-Thr216 conformer was 100% populated (Fig. 5*A*), while in the single mutant (C_12_-G191V) 86% (Fig. 5*B*), and the double glycine mutant (G188V/G191V) displayed only 64% C_12_-conformer (Fig. 5*C*). Over time, the samples hydrolyzed, and holo-peaks appeared at a nitrogen chemical shift of 104 ppm. In G188V/G191V/G195V*Sc*ACP sample, only one conformer of Thr216 was visible right from the beginning, corresponding to the holo-form, *i.e.*, at 104 ppm (Fig. 5*D*). Conceivably, C_12_-G188V/G191V/G195V*Sc*ACP gets hydrolyzed during sample preparation. Wild type C_12_-*Sc*ACP sample was stable for >48 h, C_12_-G191V*Sc*ACP single glycine mutant ∼15 h, C_12_-G188V/G191V*Sc*ACP double glycine mutant ∼9 h, and G188V/G191V/G195V*Sc*ACP (triple glycine mutant) 0 h. The stability of the C_12_-intermediates was in the order wild-type > single > double > triple glycine mutant.

### Thr 216 plays an important role in C_12-_ chain sequestration

Several threonines of helix IV displayed large perturbations in the acyl-*Sc*ACP intermediates. Helanal-Plus also predicted a partial bend at Thr 208 and Thr 212 in the *Sc*ACP structure, PDB 2UV8. To uncover the role of the two threonines in *Sc*ACP function, they were mutated to alanine. ^1^H and ^15^N chemical shift changes of T212A and T216A*Sc*ACP confirmed that the overall fold was retained in the mutant (Fig. S4, *D*, *I*, *E* and *J*).

Acylation-induced chemical shift perturbations of C_8_-, C_10_-, and C_12_-T212A*Sc*ACP were similar to wild-type *Sc*ACP (data not shown). However, T216A*Sc*ACP displayed a remarkable change in sequestration. Figure 3*E* shows the multiple overlaid ^1^H^15^N HSQC spectra for holo-, C_8_-, C_10_-, and C_12_-T216A*Sc*ACP (Fig. 3*E*). Significant chemical shift perturbations were observed for C_8_- and C_10_-T216A*Sc*ACP. However, peaks for C_12_-T216A*Sc*ACP displayed insignificant change (Fig. 3*J*). The ^1^H^15^N HSQC spectra of C_12_-216A*Sc*ACP fully overlapped with its holo-T216A*Sc*ACP, suggesting complete hydrolysis of the C_12_- chain. The molecular mass of C_12_-T216A*Sc*ACP was confirmed by ESI-MS (Fig. S2*H*). The three samples C_12_-T212A, C_12_-T216A, and C_12_-wild*Sc*ACP were prepared simultaneously, using the same *Sc*PPT, C_12_-CoA, and buffer stocks.

^1^H^15^N HSQC spectra of holo-, C_8_-, and C_10_- intermediates of T216A*Sc*ACP were compared to wild*Sc*ACP intermediates. The change in the chemical shift of holo-*Sc*ACP upon conversion to C_8_- and C_10_-wild*Sc*ACP is indicated by a black arrow, and T216A*Sc*ACP by a red arrow (Fig. S5). Interestingly, most C_10_-T216A*Sc*ACP residues (green peaks) displayed larger perturbations compared with C_10_-wild type *Sc*ACP (green peaks), shown in Figure S5, *A*–*C*. However, the C_8_- peaks of the two proteins (colored blue) were similar/comparable. Large magnitude changes of C_10_-T216A peaks probably reflect reduction in the overall size/capacity of the hydrophobic cavity upon mutation of Thr216 to Ala.

### The opening of the hydrophobic cavity of *Sc*ACP lies between helix II and loop II

**Figure 6:**
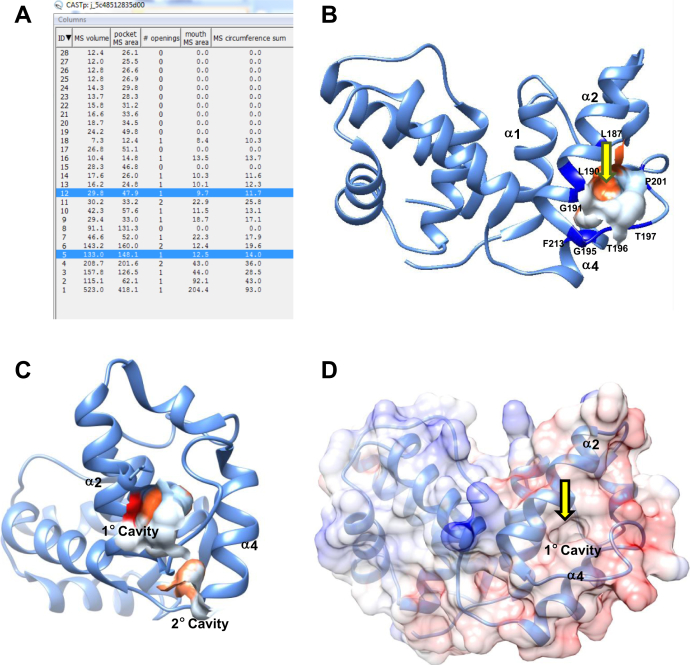
**The hydrophobic cavity of *Sc*ACP didomain (PDB****2UV8****) lies between helix II and IV.***A*, all 29 cavities predicted by CastP, based on the crystal structure of the didomain (PDB 2UV8). *B*, one of the cavities predicted by CastP #5, in compliance with NMR chemical shift perturbations appears to be the primary hydrophobic cavity. *C*, *Sc*ACP didomain, displaying the location of the primary (#5), and a second cavity (#12), present at the base of helix IV, identified based on NMR chemical shift perturbations. *D*, surface diagram of *Sc*ACP, colored based on coulombic charge. The opening of the primary cavity is shown by a *downward pointing arrow*.

CastP (Computed Atlas of Surface Topography of Proteins) 3.0 server (29) was used to identify the cavities of *Sc*ACP didomain in the crystal structure 2UV8. A total of 29 pockets were identified. As listed in Figure 6*A*, cavity #5 formed by Leu 187, Leu 190, Gly 191, Gly 195-Pro 198, Pro 201, Leu 209, and Phe 213 is in compliance with our C_8_-*Sc*ACP chemical shift perturbation data and glycine mutagenesis studies. The opening of this cavity lies between helix II and loop II. The cavity is present between the second and third turns of helix II, with Leu 187 and Gly 191 present at the mouth of the cavity (Fig. 6*B*). Notably, Leu 187 displayed large carbonyl chemical shift perturbations in our studies. We speculate that this is the opening of the primary hydrophobic cavity of *Sc*ACP, as residues forming this cavity (identified by CastP) displayed perturbations in all three intermediates, C_8_-, C_10_-, and C_12_-. Another hydrophobic cavity #12 observed at the base of *Sc*ACP is formed by Phe 194, Phe 213, Thr 216, and Phe 217. This appears to be the second cavity that harbors the C_12_- chain (Fig. 6*C*), as the aforementioned amides displayed significant perturbations upon C_12_-*Sc*ACP formation, and mutation of T216 led to the complete loss of C_12_- chain sequestration. Figure 6*D* shows the surface figure for the *Sc*ACP didomain (PDB 2UV8), displaying the opening of the cavity (shown by a downward pointing arrow). The ACP subdomain is shown as ribbons (Fig. 6*D*). CastP calculated primary cavity size (volume between the walls) was ∼134 Å^3^, and the second cavity was ∼30 Å^3^. With the help of ^3^V software, we calculated the volume of the C_8_- and C_10_- chains using PDB 2FAE (30). The calculated volume of C_8_- chain was 154 Å^3^, and C_10_- chain approx. 186 Å^3^. For the C_12_- chain, PDB 2BYZ was used, and the calculated volume was ∼228 Å^3^.

## Discussion

ACP is an essential cofactor, indispensable for fatty acid synthesis. All ACPs have a conserved four-helix bundle fold, but vary in their mechanism of function. Type I ACP does not sequester the acyl chain, while the type II ACP engulfs the acyl chain in their hydrophobic core. Fungal ACPs are an exception, as they share commonalities with both type I and type II ACP. *S. cerevisiae* ACP is a domain of a large complex and has a molecular surface similar to type I ACP. However, analogous to type II ACP, it can partially sequester the growing acyl chain in its hydrophobic core and transport it to the active site of the FAS enzymes (16). In the *Sc*FAS structure, the phosphopantetheine arm was found flipped into the active site of the ketoacyl synthase domain, suggesting conservation of a switchblade mechanism (2, 15). The molecular mechanism underlying acyl chain sequestration by *Sc*ACP and transfer to the FAS enzymes remains to be fully understood.

Our NMR chemical shift perturbation studies disclose the indispensable role of a ^188^GX_2_GX_3_G^195^ motif in *Sc*ACP function. Considering chemical shift perturbations as a measure of chain sequestration, complete loss of sequestration was observed upon mutation of the three glycines in the sequence to valine. The G191V single and G188V/G191V double mutants displayed a remarkable decrease in the chain sequestration and faster release of the acyl chain. A previous bioinformatics study on membrane proteins has shown a close link between the GX_2_GX_3_G motif and helical kinks. Nearly 62% of the time, the sequence was observed near a glycine kink, and in 48% of the cases, it contained a kink center (31). Glycine can access a wide range of phi, psi torsion angles, and thereby introduce conformational flexibility in a helix. Glycine kink plays important role in the function of membrane proteins (32, 33, 34, 35, 36). In soluble proteins as well, namely PARK7 gene, encoding DJ-1, very long chain acyl CoA dehydrogenase, cellular retinol-binding protein, thermolysin, quorum sensing master regulator of *V. cholerae* HapR, and amyloid precursor protein, kinks/bends are important for function (37, 38, 39, 40, 41, 42, 43, 44). *Sc*ACP too seems to rely on a glycine-rich ^188^GX_2_GX_3_G^195^ motif for its function. Several observations support this proposition; (a) significant amide and carbonyl chemical shift changes at the backbone of ^188^GX_2_GX_3_G^195^ sequence of helix II in the intermediates, (b) HELANAL-Plus predictions, suggesting a partial kink centred at Leu187 CO-HN Gly191 based on the crystal structure, (c) loss of acyl chain sequestration upon mutation of the three glycine in the ^188^GX_2_GX_3_G^195^ sequence to valine, and (d) faster release of the acyl chain in the glycine to valine mutants. All three glycines appear to contribute to function, as the absence of any one of them decreases the level of chain sequestration, but does not inhibit it completely. Chain protection decreased remarkably with the successive loss of glycine present in the GX_2_GX_3_G motif.

Interestingly, a helix IV residing threonine of *Sc*ACP governs sequestration of longer acyl chains. Threonine and serine both can form hydrogen bonds with the backbone of a nearby residue when present in a helical kink. The side chain -Oγ group of threonine competes with the backbone amide or carbonyl, forming an (i + 4) or (i + 3) intrahelical hydrogen bond, giving rise to a slight bend in the helix (45, 46). Ser/Thr-dependent helical kinks play important role in β2-andrenergic receptor function and also in FcγRIIB (45, 47, 48). In *Sc*ACP as well, several observations reinforce the possibility of a threonine dependent kink; large chemical shift changes at Thr 212 and Thr 216 amides upon C_12_ chain attachment, loss of C_12_- chain sequestration upon mutation of Thr 216 to Ala, Helanal-Plus analysis predicting a partial bend centered at Thr 212 in helix IV, long hydrogen bonds involving Glu 211 and Thr 212 in helix IV, and upfield carbonyl chemical shift changes of Thr 212 and Asp 213 in the C_12_- intermediate. Based on the crystal structure 2UV8, both helix II and IV of holo-*Sc*ACP are pro-kink. Carbonyl chemical shift changes suggest lengthening of the backbone hydrogen bonds near these bends upon acyl chain sequestration. We speculate that the bends intensify in the intermediates, resulting in outward movement of the post kink regions of helix II and IV, expanding cavity size, and thereby generating additional space for the acyl chain.

Combining chemical shift perturbation data with CastP analysis, we have identified the hydrophobic cavity of *Sc*ACP (PDB 2UV8) that harbors the growing acyl chain. Considering chemical shift perturbation as footprints of chain sequestration, the path traversed by the acyl chain in the cavity has been fully traced. A large cavity located near the ^188^GX_2_GX_3_G^195^ sequence of helix II and loop II is the primary cavity. The attachment of C_8_- chain induces perturbations in the residues lining this cavity. A smaller second cavity identified at the base of helix IV is surrounded by residues that display perturbations in C_10_- and C_12_- intermediates. Thus, C_8_- chain is sequestered in the primary cavity, while longer acyl chains require primary as well as the second cavity. Comparing cavity size and chain volume, at least 15% expansion of the primary cavity would be required to sequester the C_8_- chain. C_10_- chain would require a total of 30%, and C_12_- chain 40% increase in volume of the primary and second cavity combined together. Mutagenesis studies underscore the role of three helix II residing glycine, and a helix IV localized threonine in bringing about this change. In the absence of either of the residues, *Sc*ACP acyl chain protection is compromised.

Altogether, this is the first report that offers novel insights into the hydrophobic cavity of *Sc*ACP, and its unique mechanism of expansion. *Sc*ACP functions by virtue of distortions in helix II and IV that intensify upon acyl chain elongation. The conformational change induced by glycine and threonine kinks generates space to accommodate the lengthening acyl chain. For shorter chains (C_8_-), helix II perturbations are sufficient. Longer acyl chains (C_10_- and C_12_-) require the second helix to undergo a sizeable change as well. The location of the hydrophobic cavity at the carboxyl-end of helix II and IV, and its opening at an unusual position between the second and third turns of helix II, highlights unique differences between the mechanism of function *Sc*ACP and type II ACP. The overrepresentation of glycine and threonine in *Sc*ACP is a feature of membrane proteins and for the first time being reported to play a role in ACP function.

## Experimental procedures

### Cloning, expression, and purification

*S. cerevisiae* ACP (*Sc*ACP, Ala138-Leu302) and its phosphopantetheinyl transferase (*Sc*PPT, Asn1755-Gln1877) are present as domains of FAS alpha subunit (UNP P19097). The corresponding segments of the FAS gene were cloned separately in two pET28a vectors. Mutations in the ACP subdomain were generated by site-directed mutagenesis, using Phusion enzyme (Thermo Scientific). The plasmid was transformed in *E. coli* BL21 Rosetta cells. Single colonies were inoculated in 5 ml LB medium and grown overnight. The cultures were transferred to 1 l uniformly labeled [^1^H,^15^N,^13^C] M9 minimal media, containing 1 g/l ^15^NH_4_Cl. and 2 g/l ^13^C glucose. Expression of the protein was induced with 1 mM IPTG at 25 °C, for 14 to 16 h. Cells were harvested by pelleting and the lysate subjected to sonication, followed by resuspension in 20 mM Tris-HCl, pH 7.5, 100 mM NaCl. The sample was centrifuged and the supernatant loaded on a Ni^2+^-NTA chromatography column. The bound protein was eluted with 50 to 250 mM Imidazole, followed by a Superdex 200 HiLoad 16/60 size-exclusion chromatography column.

### *Sc*PPT assay

Enzyme assays were performed in 2 ml volume (20 mM Tris-HCl, pH 7.5, 100 mM NaCl, 10 mM MgCl_2_), with unlabeled or uniformly labeled [^1^H,^15^N,^13^C] 20 μM *Sc*ACP, 1 to 5 μM *Sc*PPT, and 60 μM acyl-Coenzyme A. Assays were incubated for 2 h, at 37 °C. Reaction product was qualitatively analyzed on a 10% Native-PAGE and quantitatively by ESI-MS (Thermo Scientific Orbitrap). All glycine to valine mutants, namely G188V, G191V, G195V, double mutant (G188V/G191V), and the triple mutants (G188V/G191V/G195V) *Sc*ACP required higher *Sc*PPT concentration (20–30 μM) for full conversion.

### ESI–mass spectrometry

The assay samples were passed through C_4_ Zip Tips to remove salt and the final sample eluted in 50 to 80% acetonitrile, 0.1% trifluoroacetic acid for analysis in ESI-MS (Orbitrap, Thermo Scientific). The expected molecular mass of apo-*Sc*ACP was 18,023 and holo-18,363 Da.

### NMR sample preparation and data acquisition

NMR samples for ^1^H^15^N comprised 0.3 to 0.5 mM acyl-ACP and ∼1 mM for triple resonance experiments, 20 mM Tris-HCl pH 7.5, 100 mM NaCl, 10% D_2_O, and 0.5% Sodium Azide. Two- and three-dimensional NMR experiments, namely ^1^H^15^N HSQC, HNCACB, CBCAcoNH, CCcoNH, HNcoCA, and HNCA, were acquired on a Bruker Avance III 700 MHz NMR spectrometer, equipped with a TXI probe, installed at the National Institute of Immunology, New Delhi, India. Experiments were performed at 298 K throughout. NMR data were processed on a workstation running Red Hat Enterprize Linux 5.0, using NMRPipe/NMRDraw (49). The data were multiplied by a phase-shifted sinebell apodization function in all dimensions and analyzed using Sparky (50).

^1^H^15^N HSQC spectra were acquired using 1024 data points (t2) dimension and 512 data points (t1) dimension. CBCAcoNH, HNCACB, CCcoNH experiments were collected with 1024 (t3) × 128 (t1) × 32 (t2) complex data points. Data were linear predicted in the forward direction for up to half the number of experimental points in the indirect dimension. ^15^N^13^C spectra were referenced indirectly using sodium 2, 2-dimethyl-2-silapentane-5-sulfonate (DSS) as a chemical shift standard (51).

### Data analysis

Changes in the amide chemical shift for the mutant proteins have been reported as weighted average chemical shift (Δδ_Ave_), using equation (52)

(1) $$\Delta {\text{δ}}_{\text{Ave}}={\left[{\left(\Delta {\text{δ}}_{\text{HN}}\right)}^{2}+{\left(\Delta {\text{δ}}_{\text{N}}/5\right)}^{2}\right]}^{1/2}$$

Here, Δδ_NH_ and Δδ_N_ denote chemical shift change in the proton and nitrogen dimension, respectively. A standard deviation of ±1 has been used as a cutoff and represented by the horizontal line in the figures_._

## Data availability

All data are available from the authors upon request. Please send request to Monica Sundd, monicasundd@nii.ac.in.

## Supporting information

This article contains supporting information.

## Conflict of interest

The authors declare no conflict of interest with regard to this work.
