# Supplementary material for: A GX2GX3G motif facilitates acyl chain sequestration by Saccharomyces cerevisiae acyl carrier protein
Source: J Biol Chem. 2021 Nov 9;297(6):101394. doi: 10.1016/j.jbc.2021.101394 (PMC8683515; doi:10.1016/j.jbc.2021.101394)
Supplement: Figures S1–S5 [file mmc1.pdf]

A GX_2GX_3G motif facilitates acyl chain sequestration by *Saccharomyces cerevisiae* acyl carrier protein

Garima*, Rashima Prem*, Usha Yadav and Monica Sundd§
[1]National Institute of Immunology, New Delhi-110 067, India.

SUPPORTING INFORMATION:

Figure S1. **Helix II, loop II and helix IV residues display large chemical shift changes in the intermediates.**

Figure S2. **Mass spectrometry confirms the identity of the *Sc*ACP intermediates.**

Figure S3. **Chemical shift perturbations suggest sequestration of the acyl chain in the hydrophobic cavity.**

Figure S4. **Chemical shift perturbations are largely observed near the site of mutation.**

Figure S5. **C_{10}-T216A*Sc*ACP mutant peaks display larger perturbations than C_{10}-wild*Sc*ACP.**

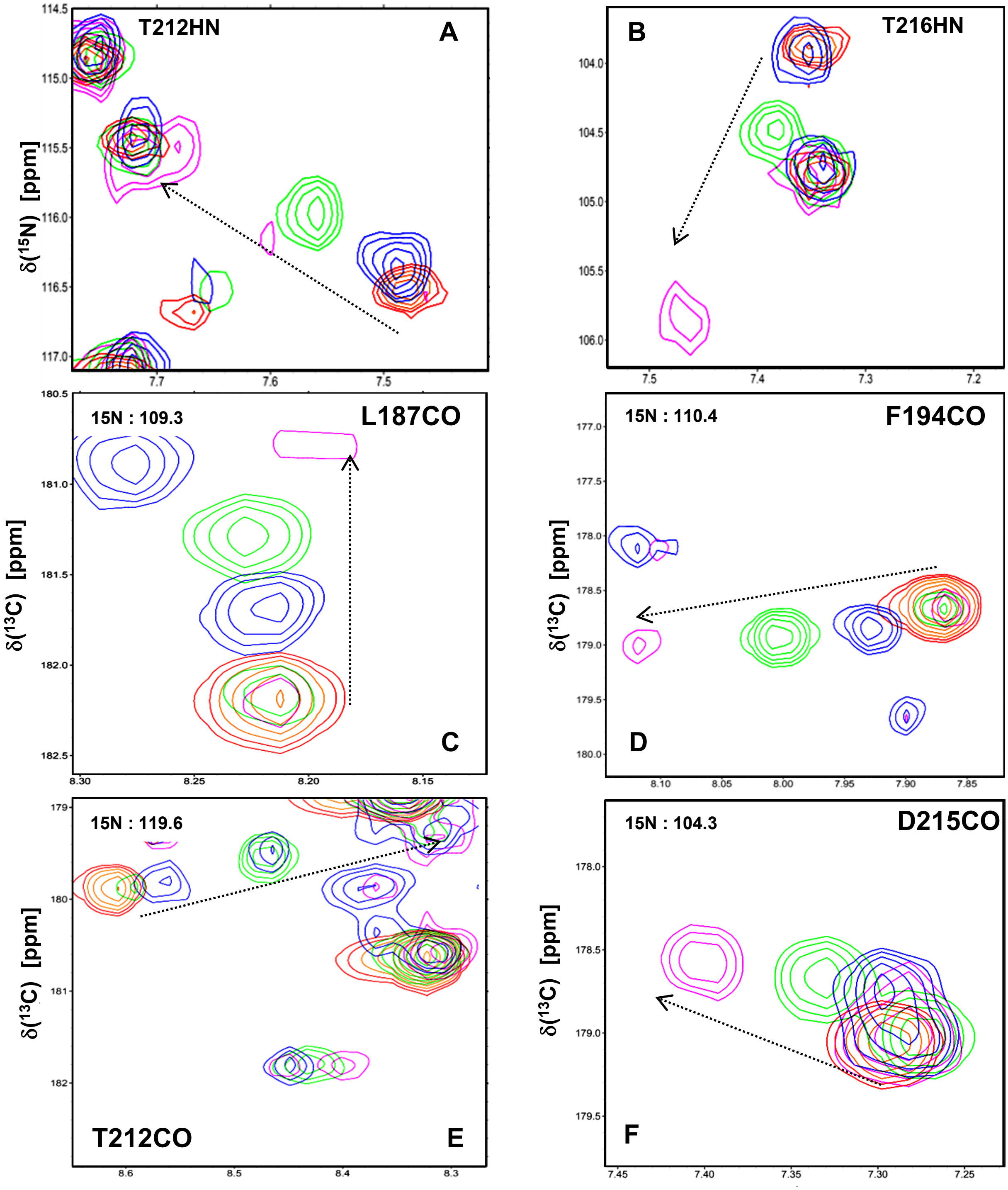

Figure S1. Helix II, loop II and helix IV residues display large chemical shift changes in the intermediates. Multiple overlaid ^{1}H^{15}N HSQC spectra for the changes in A) Thr 212, and B) Thr 216 amides in holo-, C_8-, C_{10}- and C_{12}-*Sc*ACP. Carbonyl chemical shifts changes in C) Leu 187, D) Phe 194, E) Thr 212, and F) Asp 215, observed in a ^{15}N^{13}C HNCO experiment. Peaks for holo-*Sc*ACP are colored red, C_8- blue, C_{10}- green, and C_{12}- magenta.

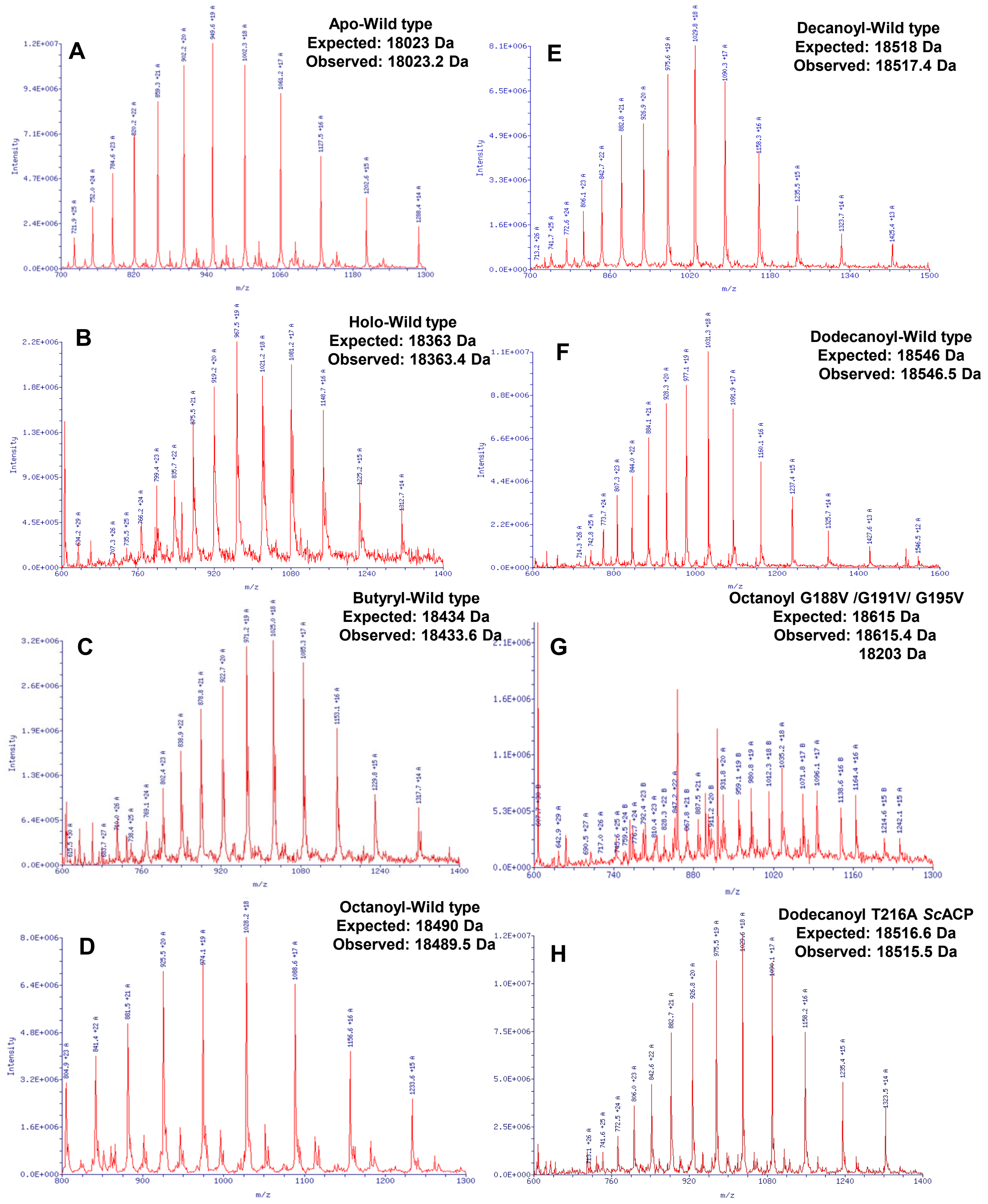

Figure S2. Mass spectrometry confirms the identity of the *Sc*ACP intermediates. ESI-MS spectra for A) apo-, B) holo-, C) butyryl-, D) octanoyl-, E) decanoyl-, F) dodecanoyl-, G) octanoyl G188V/G191V/G195V-*Sc*ACP, and H) dodecanoyl-T216A*Sc*ACP. The expected values are within ±1Da of the observed value.

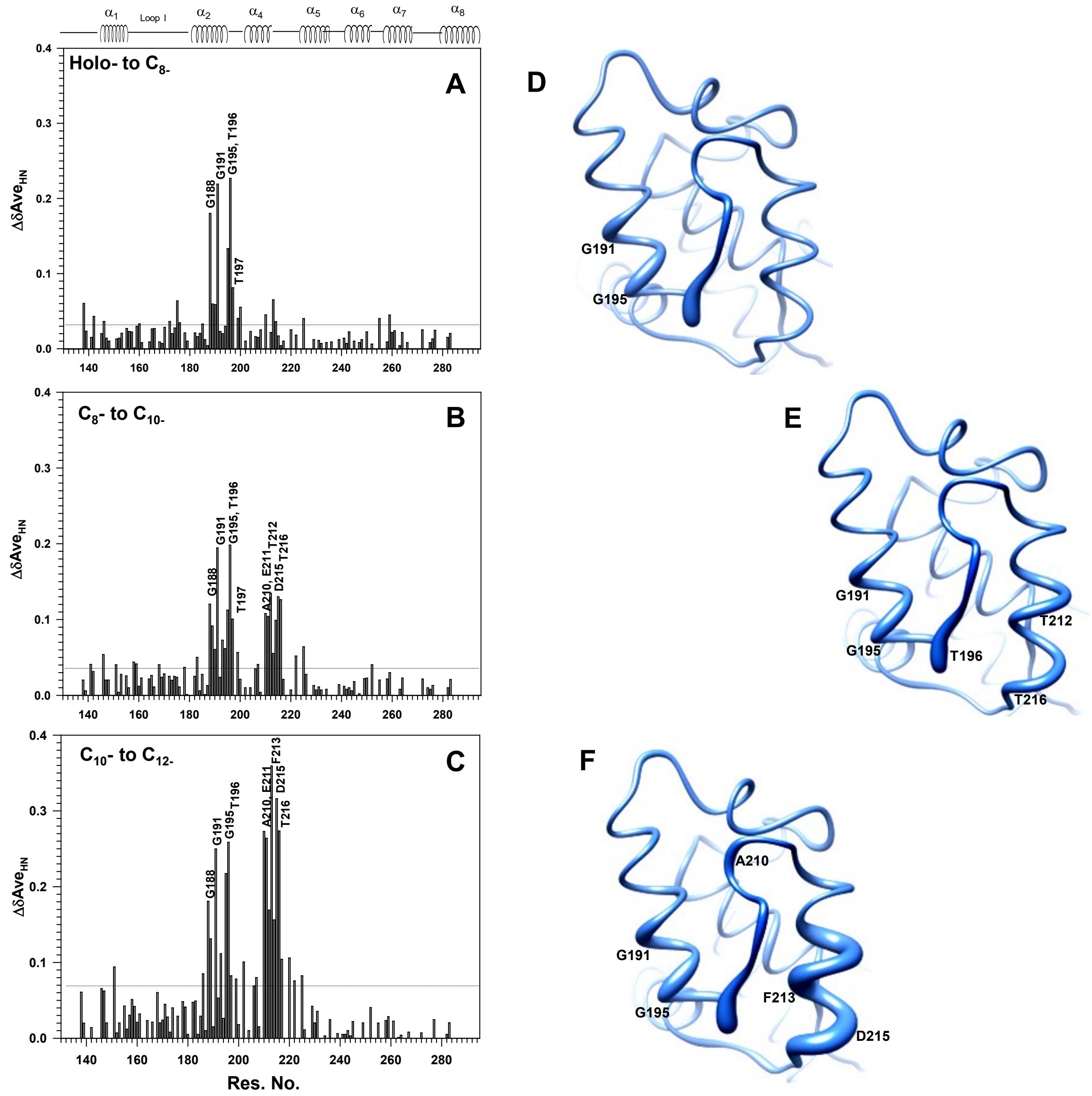

Figure S3. **Chemical shift perturbations suggest sequestration of the acyl chain in the hydrophobic cavity.** Weighted-average backbone amide chemical shift changes upon acylation in A) C_8-, B) C_{10}- and C) C_{12}- *Sc*ACP. $\Delta\delta C_8 = \delta C_8 - \delta holo$, $\Delta\delta C_{10} = \delta C_{10} - \delta C_8$, and $\Delta\delta C_{12} = \delta C_{12} - \delta C_{10}$-*Sc*ACP. A worm representation of D) C_8-, E) C_{10}- and F) C_{12}- *Sc*ACP intermediates, highlighting the changes as swellings. The thickness of the worm is directly proportional to the magnitude of chemical shift change.

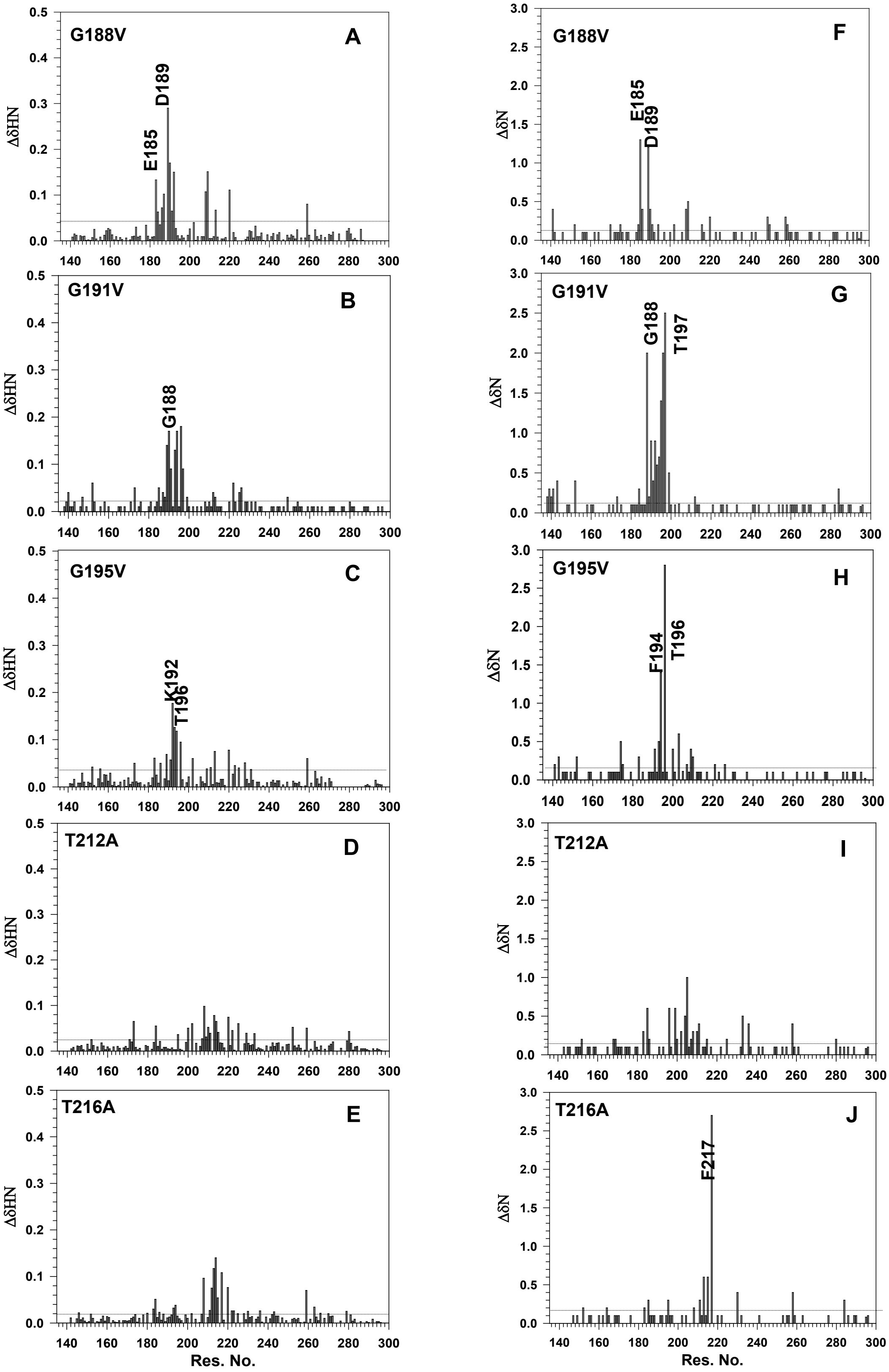

Figure S4. Chemical shift perturbations are largely observed near the site of mutation. Amide proton chemical shift changes in the mutants A) G188V, B) G191V, C) G195V, D) T212A, and E) T216A. Nitrogen chemical shift changes at the amides of F) G188V, G) G191V, H) G195V, I) T212A, and J) T216A.

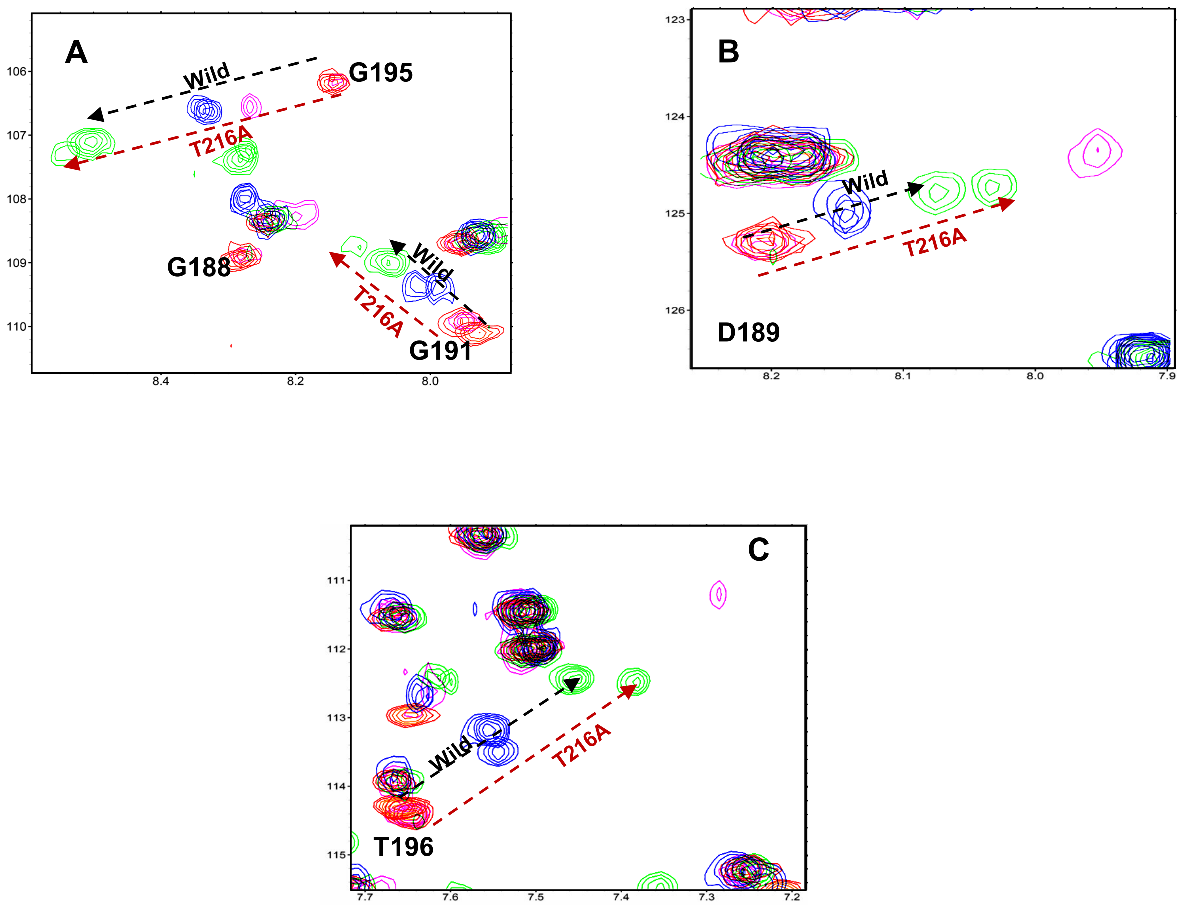

Figure S5. C_{10}-T216AScACP mutant peaks display larger perturbations than C_{10}-*Sc*ACP. Multiple overlaid $^{1}H^{15}N$ HSQC spectra displaying peaks for A) G188, G191 and G195, B) Asp 189, and C) Thr 196 in holo-, C_8-, C_{10}- and C_{12}- intermediates of wild-type *Sc*ACP, as well as T216A mutant. Holo- peaks are colored red, C_8- blue and C_{10}- green, and C_{12}- magenta.
